# Vitamin D Deficiency in Children With Vasovagal Syncope Is Associated With Impaired Circadian Rhythm of Blood Pressure

**DOI:** 10.3389/fnins.2021.712462

**Published:** 2021-08-12

**Authors:** Runmei Zou, Shuo Wang, Hong Cai, Fang Li, Ping Lin, Yuwen Wang, Cheng Wang

**Affiliations:** ^1^Department of Pediatric Cardiovasology, Children’s Medical Center, The Second Xiangya Hospital, Central South University, Changsha, China; ^2^Department of Pediatrics, Xiangya Hospital, Central South University, Changsha, China

**Keywords:** ambulatory blood pressure monitoring, vasovagal syncope, vitamin D, children, circadian rhythm

## Abstract

**Background:**

Vitamin D deficiency is associated with the risk of cardiovascular diseases. We aimed to investigate the serum vitamin D levels in children with vasovagal syncope (VVS) and explore the correlation of vitamin D status and circadian rhythm of blood pressure in VVS pediatric patients.

**Methods:**

This was a retrospective study. 130 syncopal children diagnosed with VVS were included in the study. 110 age and gender matched healthy individuals were enrolled as control. According to serum 25(OH)D levels, VVS patients were divided into vitamin D sufficient group and vitamin D deficient group. Detailed information of VVS children with vitamin D deficiency and sufficiency on demographic data, baseline laboratory testing, echocardiogram, ambulatory blood pressure monitoring, and Holter ECG recording were extracted and analyzed.

**Results:**

VVS children had a higher prevalence of vitamin D deficiency compared with healthy individuals (33.8% vs. 20.0%, *P* = 0.017). VVS patients with vitamin D deficiency had a higher rate of non-dipper blood pressure (79.5% vs. 59.3%, *P* = 0.021) and a lower value of square root of mean squared differences of successive normal to normal intervals (rMSSD) (median 107.8 vs. 141.0 ms, *P* = 0.035) compared with those with vitamin D sufficiency. Logistic regression analysis showed that non-dipper blood pressure was associated with serum 25(OH)D level [OR = 0.979, 95% CI (0.960, 0.999), *P* = 0.036].

**Conclusion:**

VVS pediatric patients had a higher prevalence of vitamin D deficiency. VVS children with vitamin D deficiency showed a higher rate of non-dipper blood pressure, suggesting that vitamin D deficiency is correlated with impaired circadian rhythm of blood pressure.

## Introduction

Vasovagal syncope (VVS), the most common cause of syncope in pediatric patients (Villafane et al., 2021), presents with an inability to maintain postural tone and very brief loss of consciousness, followed by spontaneous recovery without neurologic sequelae. VVS is related to emotional stress, prolonged standing, postural changes, pain, and a crowded environment. The mechanism of VVS remains incompletely revealed. Excessive activation of vagal tone via dysregulation of Bezold-Jarisch reflex, together with a decreased sympathetic tone, contributes to the pathogenesis of VVS (Stewart et al., 2017). It is thought that VVS is a benign condition and does not lead to mortality so far. However, frequent syncope increases the risk of injuries and reduces the quality of life significantly (Grimaldi Capitello et al., 2016).

Vitamin D is a group of fat-soluble molecules, playing a vital role in regulating numerous physiological processes in addition to calcium and phosphorus homeostasis. Though the effect of vitamin D deficiency and cardiovascular disease is controversial, observational studies reported an association between vitamin D deficiency and the risk of cardiovascular disease (Latic and Erben, 2020; Xie et al., 2020). Adolescents suffering from severe fatigue and/or orthostatic intolerance exhibited vitamin D deficiency (Antiel et al., 2011). The prevalence of hypovitaminosis D (25-hydroxyvitamin D ≤ 50 nmol/L) was 22% in adolescent patients with excessive postural tachycardia, while it was 14% in healthy individuals (Saintonge et al., 2009; Antiel et al., 2011). A previous study reported that syncopal adult patients had a lower level of vitamin D when compared to healthy individuals, especially for VVS patients diagnosed by head-up tilt test (HUTT) (Usalp et al., 2020). For pediatric patients with VVS, vitamin D levels were significantly decreased compared with healthy controls, and correlation analysis suggested that serum 25-hydroxyvitamin D levels may be associated with rMSSD values (Zhang et al., 2021). These results indicated an important role of vitamin D in VVS. However, data on the correlation between serum vitamin D level and VVS in pediatric patients need to be further explored.

In the study, we aimed to investigate the vitamin D level in pediatric patients with VVS and compare the clinical characteristics of VVS pediatric patients with vitamin D sufficiency and vitamin D deficiency to explore the correlation of vitamin D status and circadian rhythm of blood pressure.

## Study Population and Methods

### Study Population and Data Collection

This was a retrospective study and data were collected from patients who complained of recurrent syncope and visited Syncope Ward, Children’s Medical Center, The Second Xiangya Hospital, Central South University between January 2018 and December 2019. The sample sizes was calculated according to the following formula: *n*_1_ = *n*_2_ = 2[(t_α_ + t_β_)s/δ]^2^ [α = 0.05 (two-side), β = 0.10, *s* = 25, δ = 10]. Two researchers who were blinded to the study protocol collected the data separately. Patients with neurological, cardiogenic, and psychological diseases were excluded. 130 syncopal patients (4–15 years old) diagnosed with VVS by HUTT were recruited in the study. Detailed information of VVS patients on demographic data, baseline laboratory testing, echocardiogram, ambulatory blood pressure monitoring, and Holter ECG recording were reviewed and extracted. 110 age and gender matched healthy individuals (4–16 years old) without malnutrition were enrolled as control. These children came to visit our child health clinic for physical examination between January 2018 and December 2019. VVS patients were divided into two groups as those with vitamin D levels above or below 50 nmol/L (Holick et al., 2011).

### Methods

#### HUTT Protocol

The protocol of HUTT had been conducted according to the previous study (Wang et al., 2018). The HUTT was approved by the Ethics Committee of The Second Xiangya Hospital, Central South University [Ethical Audit No. Study 012 (2014)]. Informed consent was issued by all the subjects directly or their guardians prior to the test. Subjects were asked to lay still for 10 min, and then basic heart rate (HR), blood pressure (BP), and ECG were recorded. Subjects were tilted at 60° head upward. HR, BP, and ECG were recorded continuously until either 45 min duration or development of syncope or intolerable near syncope symptoms. If syncope occurred, subjects were rapidly put in the supine position. If syncope or presyncope did not occur, tilted posture was maintained, subjects were sublingually medicated with nitroglycerin (4–6 μg/kg, maximum ≤300 μg), and HR, BP, and ECG were recorded until for 20 min or syncope or presyncope occurred.

Vasovagal syncope was defined as the development of syncope or presyncope accompanied by hypotension (systolic BP ≤ 80 mmHg in children, and/or diastolic BP ≤ 50 mmHg, or over 25% decrease in mean BP), bradycardia (<75 bpm in children between 4 and 6 years old, <65 bpm in children between 6 and 8 years old, and <60 bpm in children above 8 years old), or cardiac arrest >3 s (Wang et al., 2018).

#### Ambulatory Blood Pressure Monitoring (ABPM)

Twenty-four-hour BP data were obtained from a 24-h ABPM device with a suitably sized cuff (ABPM6100, Welch Allyn, United States). The subjects were asked to continue their regular activity and sleep schedule, but to stay motionless during the period of BP measurement. BP data were measured every 15 min at daytime and every 30 min at nighttime. Daytime BP was defined as the mean of BP values measured from 6:00 to 22:00 h, whereas nocturnal BP was defined as the mean of BP values measured from 22:00 to 6:00 h of the next day. For acquiring high-quality ABPM recordings, ABPM data should meet the following conditions: at least 70% valid recordings, at least 20 daytime and 7 nighttime valid recordings, and at least one valid recording for each hour. The nocturnal BP dipping rate was defined based on systolic blood pressure (SBP) data as follows: (daytime SBP−nighttime SBP)/(daytime SBP) × 100. Nocturnal dipping status was classified into two categories according to the dipping rate in the study, dipper (≥10% but <20%) and non-dipper (≥0% but <10%) (Williams et al., 2018).

#### Heart Rate Variability (HRV)

Twenty-four-hour electrocardiogram was recorded by 12-channel 24 h Holter devices (TLC 4000 Dynamic ECG Recording Analyzer, Kangtai, China). The sampling rate was 10,000 Hz and the response band 0.05 to 60 HZ. HRV was analyzed by using an automatic Holter analysis system (TLC4000, Kangtai, China). Each RR interval was manually screened before the analysis. Only normal to normal (NN) beats were used for analysis with intervals. Interfering signals were automatically excluded by the standardized QRS peak detection of the analysis system. The time domain parameters, including standard deviations for all NN heart rate intervals over 24 h (SDNN), the standard deviation of the averages of NN intervals calculated over 5-min periods of the entire recording (SDANN), percentage of differences between adjacent RR intervals that are >50 ms (PNN50), and square root of mean squared differences of successive NN intervals (rMSSD) were analyzed (Wang et al., 2019).

### Statistical Analysis

Statistical analysis was performed by SPSS 24.0 (IBM Corp., Armonk, NY, United States). Continuous variables for data following normal distribution were described as mean ± SD and analyzed by Student *t*-tests. Continuous variables for data not following normal distribution were expressed as the median with interquartile range (IQR) and analyzed using the Mann-Whitney *U* test. Categorical data were described using frequencies and percentages. The χ^2^ test or Fisher’s exact test was used to compare the differences of the categorical variables. Association for serum 25(OH)D level and rMSSD was analyzed by Spearman correlation coefficient. For the logistic regression model, variables including age, gender, body mass index, serum 25(OH)D level, and rMSSD that were associated with non-dipper BP were enrolled. *P*-value < 0.05 was considered to be statistically significant differences.

## Results

A total of 130 VVS patients [mean age 10.7 ± 2.5 years, 69 females (53.1%)] and 110 healthy individuals [mean age 10.3 ± 2.3 years, 50 females (45.5%)] were included in the study. There were no significant differences in age, gender, and body mass index (BMI) between healthy individuals and VVS patients (all *P* > 0.05). Serum 25(OH)D level was significantly lower in patients with VVS compared with healthy individuals (59.8 ± 21.4 vs. 65.9 ± 19.2 nmol/L, *P* = 0.022). VVS patients had a higher rate of vitamin D deficiency compared with healthy individuals (33.8% vs. 20.0%, *P* = 0.017) (Table 1).

**TABLE 1:** Clinical characteristics of healthy individuals and vasovagal syncope patients.

**Variables**	**Control group (*n* = 110)**	**VVS group (*n* = 130)**	***P***
Male, *n* (%)	60 (54.5)	61 (46.9)	0.239
Female, *n* (%)	50 (45.5)	69 (53.1)	
Age (years)	10.3 ± 2.3	10.7 ± 2.5	0.143
BMI (kg/m^2^)	17.8 ± 4.1	17.3 ± 2.9	0.290
Serum 25(OH)D (nmol/L)	65.9 ± 19.2	59.8 ± 21.4	0.022
25(OH)D deficiency rate, *n* (%)	22 (20.0)	44 (33.8)	0.017

*BMI, body mass index.*

According to serum vitamin D level, VVS patients were divided into two groups, vitamin D sufficient group with serum 25(OH)D level greater than or equal to 50 nmol/L and vitamin D deficient group with serum 25(OH)D level below 50 nmol/L. As shown in Table 2, There was no significant difference between the two groups regarding gender, age, BMI, and laboratory findings including white blood counts, hemoglobin, platelets, serum thyroid-stimulating hormone, glucose, alanine aminotransferase, aspartate aminotransferase, serum creatinine, and uric acid (all *P* > 0.05).

**TABLE 2:** Demographic characteristics and laboratory findings of patients with vasovagal syncope.

**Variables**	**25(OH)D sufficient group (*n* = 86)**	**25(OH)D deficient group (*n* = 44)**	***P-*value**
Male, *n* (%)	44 (51.2)	17 (38.6)	0.176
Female, *n* (%)	42 (48.8)	27 (61.4)	
Age (years)	10.7 ± 2.5	10.7 ± 2.4	0.971
BMI (kg/m^2^)	17.3 ± 2.5	17.2 ± 3.5	0.855
WBC (×10^9^/L)	6.1 ± 1.5	5.58 ± 0.9	0.081
HGB (g/L)	126.1 ± 12.5	122.8 ± 24.8	0.415
PLT (×10^9^/L)	278.0 ± 65.1	279.5 ± 64.0	0.917
TSH (uIU/ml)	2.8 ± 1.4	3.2 ± 1.8	0.469
Glucose (mmol/L)	4.5 ± 0.7	4.5 ± 0.6	0.978
ALT, M(IQR), U/L	10.4 (8.7, 14.4)	10.7 (7.1, 12.7)	0.487
AST, M(IQR), U/L	21.5 (17.3, 25.7)	20.3 (15.4, 26.5)	0.207
SCr (μmol/L)	41.7 ± 10.3	43.7 ± 9.6	0.422
UA (μmol/L)	301.4 ± 81.6	299.9 ± 87.3	0.936

*BMI, body mass index; WBC, white blood cell; HGB, hemoglobin; PLT, platelet; TSH, thyroid stimulant hormone; ALT, alanine aminotransferase; AST, aspartate aminotransferase; SCr, serum creatinine; UA, uric acid; IQR, inter quartile range.*

There was no significant difference between VVS patients with sufficient and deficient vitamin D when compared on ejection fraction, fraction shortening, daytime and nocturnal systolic blood pressure, daytime and nocturnal diastolic blood pressure, and mean heart rate (all *P* > 0.05). Parameters of HRV, SDNN, SDANN, and PNN50 had no significant differences between the two groups. However, rMSSD of VVS children with vitamin D deficiency was lower than those with vitamin D sufficiency (median 107.8 vs. 141.0 ms, *P* = 0.035) (Table 3). Spearman correlation analysis demonstrated that rMSSD was not correlated with 25(OH)D levels (*r* = 0.166, *P* = 0.072). VVS patients with vitamin D deficiency had a higher rate of non-dipper blood pressure compared with those with normal vitamin D levels (79.5% vs. 59.3%, *P* = 0.021) (Table 3). Serum 25(OH)D level, rMSSD, age, gender, and BMI were enrolled for logistic regression analysis, results showed that non-dipper blood pressure was associated with serum 25(OH)D level [OR = 0.979, 95% CI (0.960, 0.999), *P* = 0.036], but not related to rMSSD, age, gender, and BMI (all *P* > 0.05) (Table 4).

**TABLE 3:** Electro-cardiological indictors of patients with vasovagal syncope.

**Variables**	**25(OH)D sufficient group (*n* = 86)**	**25(OH)D deficient group (*n* = 44)**	***P***
EF (%)	66.6 ± 3.7	66.6 ± 3.8	0.987
FS (%)	36.2 ± 4.0	36.8 ± 3.3	0.455
dSBP (mmHg)	107.3 ± 16.4	103.9 ± 23.4	0.354
dDBP (mmHg)	60.7 ± 6.7	60.6 ± 5.0	0.965
nSBP (mmHg)	98.8 ± 15.8	100.7 ± 10.4	0.489
nDBP (mmHg)	51.8 ± 6.6	53.0 ± 5.0	0.305
Non-dipper BP, *n* (%)	51 (59.3)	35 (79.5)	0.021
mHR (beats/min)	80.5 ± 8.3	79.4 ± 9.4	0.538
SDNN, M(IQR), ms	164.2 (141.3, 191.6)	153.0 (133.5, 189.7)	0.248
SDANN (ms)	146.5 (122.1, 181.9)	138.1 (109.8, 171.9)	0.266
rMSSD, M(IQR), ms	141.0 (96.5, 193.7)	107.8 (79.8, 146.5)	0.035
PNN50 (%)	38.1 ± 15.3	35.1 ± 15.6	0.330

*EF, ejection fraction; FS, fraction shortening; dSBP, daytime systolic blood pressure; dDBP, daytime diastolic blood pressure; nSBP, nocturnal systolic blood pressure; nDBP, nocturnal diastolic blood pressure (Daytime BP was defined as the mean of BP values measured from 6:00 to 22:00 h, whereas nocturnal BP was defined as the mean of BP values measured from 22:00 to 6:00 h of the next day); mHR, mean heart rate; SDNN, standard deviations for all normal to normal (NN) heart rate intervals over 24 h; SDANN, standard deviation of the averages of NN intervals calculated over 5-min periods of the entire recording; rMSSD, square root of mean squared differences of successive NN intervals; PNN50, percentage of differences between adjacent RR intervals that are >50 ms; M, median; IQR, inter quartile range.*

**TABLE 4:** Logistic regression analysis between non-dipper blood pressure and serum 25(OH)D level, rMSSD, gender, age, and BMI.

**Variables**	**Beta**	**SE**	**Ward**	***P*-value**	**OR**	**95% CI of OR**
25(OH)D level	–0.021	0.010	4.408	0.036	0.979	0.960, 0.999
rMSSD	0.004	0.003	1.762	0.184	1.004	0.998, 1.009
Gender	–0.125	0.409	0.094	0.760	0.882	0.395, 1.968
Age	–0.090	0.093	0.947	0.330	0.914	0.762, 1.096
BMI	–0.082	0.078	1.097	0.295	0.921	0.790, 1.074

*BMI, body mass index; rMSSD, square root of mean squared differences of successive normal to normal intervals.*

## Discussion

In the present study, we found that serum 25(OH)D level was lower in VVS patients than that of healthy individuals. VVS patients had a higher prevalence of vitamin deficiency. VVS patients with vitamin D deficiency exhibited a higher rate of non-dipper blood pressure compared with those with normal vitamin D levels. Logistic regression analysis suggested that non-dipper blood pressure was closely associated with serum vitamin D level.

Vasovagal syncope is caused by an abnormal reaction of the autonomic system to various stimuli, such as a triggering event or posture change. Prolonged standing or posture change leads to decreased venous return, which results in inadequate ventricular filling and vigorous cardiac contraction. Mechanoreceptors (C fibers) located preferentially in the inferolateral wall of the left ventricle are activated, resulting in hypotension and paradoxical bradycardia due to increased activity of inhibitory receptors and consequent parasympathetic hyperactivity (Medow et al., 2008).

In our study, VVS patients exhibited a higher rate of vitamin D deficiency than healthy individuals. The reason why VVS patients had vitamin D deficiency may be concluded in several mechanisms. Firstly, vitamin D is important in maintaining cardiac autonomic function. The low level of vitamin D (<50 nmol/L) caused cardiac autonomic dysfunction by repressing vagal activity and increased the risk of cardiovascular diseases (Mann et al., 2013). Vitamin D supplementation significantly improved HRV parameters of healthy individuals with vitamin D deficiency (Mann et al., 2014; Dogdus et al., 2019). Secondly, the active form of vitamin D plays a critical role in the proliferation of vascular smooth muscle cells and endothelial cells. Vitamin D regulates blood pressure by acting on vascular smooth muscle cells and endothelial cells (de la Guia-Galipienso et al., 2021). Endothelium-derived nitric oxide-evoked dilation was decreased in arteries from vitamin D deficient rats (Tare et al., 2011), suggesting vitamin D deficiency prompts endothelial vasodilator dysfunction. Vitamin D deficiency may result in syncope by deterioration of vascular function. Moreover, vitamin D deficiency may disturb neuronal conduction in the baroreflex mechanism (Naveilhan et al., 1996), which is closely related to the onset of syncope.

During the course of 24 h, BP behavior exhibits a trend characterized by lower BP at nighttime than at daytime. For healthy individuals, BP at nighttime decreases at 10–20% and with a peak in the early morning. Previous studies indicated that abnormal diurnal blood pressure profile is associated with subclinical target organ damage, cardiovascular events, and mortality (Gong et al., 2019). In a previous study of newly diagnosed hypertension, patients with vitamin D deficiency had a higher nighttime SBP and non-dipper SBP pattern compared to patients with sufficient vitamin D (Cakal et al., 2021). In our research, there was no difference in daytime and nocturnal BP between VVS children with sufficient and deficient vitamin D. However, children with vitamin D deficiency had a higher rate of non-dipper blood pressure. We further performed logistic regression analysis, and the results showed that non-dipper blood pressure was associated with serum vitamin D levels. Vitamin D is important in maintaining cardiac autonomic function. Vitamin D deficiency may disturb autonomic function, resulting in the disorder of blood pressure circadian rhythm.

Heart rate variability is a non-invasive and simple method for the assessment of cardiac autonomic function (Lahiri et al., 2008). It consists of time-domain and frequency-domain analysis. In the time-domain analysis, SDNN indicates the general measurement of autonomic nervous system balance, rMSSD and PNN50 indicate the parasympathetic activity of heart. In the frequency-domain analysis, HF is under the influence of the parasympathetic nervous system, while LF is modulated by both parasympathetic and sympathetic nervous systems. Previous studies demonstrated that HRV parameters as SDNN, SDANN, rMSSD, PNN50, and HF were significantly decreased in healthy individuals with vitamin D deficiency compared with those with sufficient vitamin D (Canpolat et al., 2015; Dogdus et al., 2019). These results suggested that vitamin D deficiency was significantly associated with impaired cardiac autonomic functions in healthy individuals. In children and adolescents with VVS, vitamin D deficiency exhibited a lower rMSDD value compared to the vitamin D sufficient group (Zhang et al., 2021). In our study, rMSDD value was decreased in VVS children with vitamin D deficiency compared to those with vitamin D sufficiency, which was consistent with the previous study. The result demonstrates that vitamin D deficiency may be related to the decreased parasympathetic activity of the heart, paradoxically activates sympathetic activity, which makes the patients more prone to trigger the Bezold-Jarisch reflex and suffer syncope. However, Spearman analysis indicated that rMSSD value was not correlated with serum vitamin D levels in our study. More studies should be performed to explore the relation between vitamin D levels and HRV in VVS children.

Our study is a retrospective study, and there are some limitations. First, vitamin D is regulated by calcium, phosphate, fibroblast growth factor 23 (FGF23), and parathyroid hormone (PTH) levels. However, data of calcium, phosphate, FGF23 and PTH levels were not tested in the study. Besides, data on ambulatory blood pressure for healthy individuals were not obtained in our study. The circadian rhythm of blood pressure for healthy individuals with vitamin D deficiency was not analyzed.

In spite of its limitation, the findings of our research showed that VVS pediatric patients had a higher prevalence of vitamin D deficiency compared with healthy children. VVS pediatric patients with vitamin D deficiency showed a higher rate of non-dipper blood pressure, suggesting little blood pressure variability in these patients. Arterial blood pressure variability could detect early autonomic alterations (Genovesi et al., 2008). Analysis of arterial blood pressure variability may be helpful for further assessing autonomic function of VVS children with vitamin D deficiency. Furthermore, vitamin D deficiency may be correlated with impaired circadian rhythm of blood pressure in VVS pediatric patients. Whether vitamin D supplementation is helpful to improve the circadian rhythm of blood pressure and autonomic function should be further explored.

## Data Availability Statement

The raw data supporting the conclusions of this article will be made available by the authors, without undue reservation.

## Ethics Statement

The studies involving human participants were reviewed and approved by the Ethics Committee of The Second Xiangya Hospital, Central South University [Ethical Audit No. Study 012 (2014)]. Written informed consent to participate in this study was provided by the participants’ legal guardian/next of kin.

## Author Contributions

RZ and CW had primary responsibility for the protocol development, patient enrollment, data collecting, preliminary data analysis, and writing the manuscript. SW assisted with data analysis and critical revision for important content, and edited the draft. HC, FL, PL, and YW took the responsibility to complete the head-up tilt test and ambulatory blood pressure monitoring. CW supervised the design and execution of the study, checked the data analysis, and contributed to a final approval of the manuscript submitted. All authors have read and approved the final manuscript and assumed full responsibility for its contents.

## Conflict of Interest

The authors declare that the research was conducted in the absence of any commercial or financial relationships that could be construed as a potential conflict of interest.

## Publisher’s Note

All claims expressed in this article are solely those of the authors and do not necessarily represent those of their affiliated organizations, or those of the publisher, the editors and the reviewers. Any product that may be evaluated in this article, or claim that may be made by its manufacturer, is not guaranteed or endorsed by the publisher.
